# Give PrEP a chance: moving on from the “risk compensation” concept

**DOI:** 10.1002/jia2.25351

**Published:** 2019-08-30

**Authors:** Daniela Rojas Castro, Rosemary M Delabre, Jean‐Michel Molina

**Affiliations:** ^1^ Coalition PLUS Community‐based Research Laboratory Pantin France; ^2^ Aix Marseille Univ INSERM, IRD, SESSTIM Sciences Economiques & Sociales de la Santé & Traitement de l'Information Médicale Marseille France; ^3^ Department of Infectious Diseases Hôpital Saint‐Louis Assistance Publique Hôpitaux de Paris Paris France; ^4^ INSERM, UMR 941 Université de Paris Diderot Paris 7 Sorbonne Paris Cité Paris France

**Keywords:** bio‐behavioural prevention, PrEP, risk compensation, sexual behaviour, sexually transmitted infections, HIV

## Abstract

**Introduction:**

While bio‐behavioural interventions (BIs) for sexually transmitted infections (STIs) and HIV prevention have shown their effectiveness (e.g. treatment for syphilis, HPV vaccination or pre‐exposure prophylaxis [PrEP]), they have also aroused major concerns regarding behavioural changes that could counteract their benefit. Risk compensation (RC) fears concerning BIs in the HIV/STIs prevention field are intimately linked to representations, judgements and social control on sexual behaviour. With an increasing number of PrEP studies describing a rise in STIs due to RC, this paper argues for a shift away from the focus on RC and proposes a more constructive approach to respond to the needs of people living with HIV and populations most at risk.

**Discussion:**

The concept of RC, stemming from road safety and derived from economic theory, relies on rational theoretical models of human behaviour. Although widely applied in several contexts its use has been reasonably questioned. Major methodological issues regarding RC have been raised within HIV/AIDS literature. Although behavioural changes (e.g. condomless sex and number of sexual partners) are often erroneously assimilated with RC, there is no evidence that behavioural changes have undermined the effectiveness of previous and current BIs. Still, PrEP has not escaped RC concerns. Increases in condomless sex within the context of growing uptake of PrEP signals a continued need for integrated and innovative HIV and STI prevention strategies and a comprehensive sexual health approach. Routine HIV/STI testing, peer‐led counselling, and identification of sexual health needs within the PrEP model of care could become a gold standard in the sexual health field for all populations.

**Conclusions:**

RC remains a frequent argument against the availability and provision of prevention methods for vulnerable populations. Individuals should be able to benefit from the full panel of BIs options available, to find and adapt methods according to their needs. Current, past and future PrEP users, with other stakeholders, may provide valuable insight into innovative solutions and programmes to control HIV and other STIs.

## 1 Introduction

In 2015, in spite of strong evidence of the efficacy of pre‐exposure prophylaxis (PrEP) to prevent HIV infection 1, 2, 3, 4 and WHO recommendations 5, a rebuttal to the Lancet HIV editorial “PrEP: why are we waiting?” stated that decision‐makers lacked information regarding the “normative aspects” of PrEP use 6. More precisely, they explained that the main reason for not implementing this bio‐behavioural intervention (BI) was lack of information regarding “people's own responsibility to use a condom, the relevance of being free of fear of HIV infection when having sex, and the relative importance of preventing HIV versus a possible rise in other sexually transmitted diseases because of reduced condom use” 6. This quote makes explicit important points that have overshadowed PrEP and other BI: moral judgements on sex and HIV prevention as a means of controlling sex 7, 8, 9.

What the PrEP example shows is nothing new. In the last decades, other prevention tools were all met with caution as they could possibly induce behavioural changes leading to an increased risk and consequently counteract the benefit of the prevention tool in question: the oral contraceptive pill in the 1950s 10, 11, treatment for syphilis in the 1960s 12 and 1970s 13, 14, needle exchange programmes for injecting drug users 15, 16, 17, the morning‐after pill 18, and more recently HPV vaccination 19, 20, 21. Although different BI for HIV prevention have shown their effectiveness (e.g. condoms, male circumcision, highly‐active antiretroviral treatment (HAART), post‐exposure prophylaxis (PEP), treatment as prevention (TasP) and pre‐exposure prophylaxis (PrEP)), each and every one has aroused concerns regarding “risk compensation” (RC) 22, 23, 24, 25. The HIV/AIDS field has scarcely challenged the use of the RC concept 26 at the expense of focusing on other positive aspects of BI such as increased quality of (sexual) life, empowerment to discuss safer sex and to disclose HIV status, reduced fear of transmitting or getting HIV, or the possibility to re‐engage in sexual activity after an HIV diagnosis, to name a few 27, 28, 29.

At the start of the epidemic, sexually transmitted infections (STIs) were already present and a health concern 30. Most likely due to its fatal nature and lack of treatment, demanding specific medical interventions and innovations, HIV/AIDS was treated separately from other STIs. Evidence that STIs facilitate HIV transmission led to recognition of an “epidemiological synergy” between HIV and other STIs, thus leading to calls for prevention programmes and strategies that addressed both HIV and other STIs 31, 32. Whereas some prevention methods such as condoms provide protection against HIV and other STIs, other “no barrier” HIV prevention strategies such as TasP and PrEP have changed the scene.

In the context of an increasing number of PrEP studies describing a rise in STIs due to “RC,” this paper provides a critical view of the origin, use and consequences of this concept in the HIV prevention field and argues for a shift away from the focus on RC. In a time when more effort is needed to reduce the number of new infections among key populations (KP) and their sexual partners 33, and STIs are a health concern, we propose a more constructive approach that responds to the needs of people living with HIV (PLHIV) and most‐at‐risk populations.

## 2 Discussion

### 2.1 Is RC a pertinent and valid framework?

Although RC has been used interchangeably with “disinhibition” in scientific literature, these are in fact two different concepts 14. *Disinhibition* refers to the lowering or absence of self‐restraint to avoid risk 14, 34; for example when an inebriated person is aggressive or engages in sexual risk behaviour (SRB) because he/she no longer cares about the risk 35. *Risk compensation* is related to the “risk equilibrium” which is defined as “a system in which individuals accept a certain level of subjectively estimated (or perceived) risk to their health in exchange for benefits they expect to receive from (an) activity” 36.

Since most of the literature regarding BI refers to RC, it is worth focusing on the origins of this widely used concept. The National Highway Traffic Safety Administration (USA), with the goal of preventing road injuries, issued in 1968 29 Federal Motor Vehicle Safety Standards (FMVSS) regarding features such as seat belts. In 1975, economist Sam Peltzman, evaluated FMVSS with the perspective that since safety is an exchangeable “good,” individuals would exchange safety for “driving intensity” if the car is safer than expected 37. His results, since proven to be erroneous 38, led to the conclusion that security standards had no effect on overall traffic fatalities and increased pedestrian deaths. Decades of debates on these results, but also on others such as those showing seat belt laws were not effective 39, 40, 41, introduced RC as a plausible framework to understand road safety despite experiments unable to provide useful evidence and evaluation contaminated by poor data and uncontrolled factors 42.

There exist well‐established psychosocial theories and models to approach the behavioural change in relation to health, such as, amongst others, the theory of reasoned action/planned behaviour 43, 44, 45, 46, the transtheoretical model of behaviour change 47 or the information‐motivation‐skills model 48, 49, 50. However, the road safety field has focused on so‐called “risk models,” such as the “Threat‐avoidance model” 51, the “Model of drivers’ decision making and behaviour” 52 or the “Risk Homeostasis Model” 53, in which the risk concept plays a major role. The concept of risk homeostasis or RC described in 1982 claims that human behaviour falls under the same mechanism as a thermostat 54. Thus, interventions to prevent car accidents, or the use of helmets by bicycle riders 55, would not be useful since individuals would change their behaviour so that their level of risk stays constant 56, 57. The RC concept relies on rational theoretical models of human behaviour, derived from economic theory, that have been widely criticised 58, 59, 60, nevertheless it has attracted great attention 61. Otherwise, literature has shown that seat belts and helmets do not lead to behavioural changes leading to a risk increase and are, undoubtedly, effective 60, 62, 63.

Methodological issues regarding RC have been also raised within HIV/AIDS literature 64. To accurately claim that a BI leads to an increased risk for HIV, a randomized control trial would have to compare a group believing that the intervention would reduce risk with another group believing that the intervention would not reduce risk 22. Because of ethical issues, this design is not a viable option 64. Other methodological considerations have been drawn 23: (1) studies are mostly focused on behavioural measures, failing to account for the possibility that changes in attitudes or risk perceptions (essential to the RC theory) may occur before behaviour change; (2) timing in the change of attitudes and behaviour is important but not always clear; condomless sex (CLS) can precede “optimistic attitudes” regarding HIV exposure; (3) some studies did not find that change in behaviour led to risk increase 2, 65, 66, 67, 68, 69; (4) even if changes in behaviour or risk perception are observed they will likely not undermine the high effectiveness of the prevention strategy 23; (5) interventions are not considered from a community level, therefore are limited to an individual approach 23.

### 2.2 Evidence of changes in sexual behaviour or evidence of “risk compensation”?

Despite the emergence of various forms of BI, strategies such as male circumcision 25 and condom promotion were suspected of engendering RC 70. However, these strategies did not induce enough behavioural changes to have an impact on their effectiveness 71, 72. The advent of HAART in 1996 led to obvious beneficial clinical effects. HIV was no longer perceived as a life‐threatening disease 73, 74, 75, generating fears of unintended effects on sexual behaviour 76, 77 and on the incidence of STIs 78. Increasing public information on how an undetectable viral load reduces the level of infectiousness of HIV‐positive individuals 65, which was then confirmed in the “Swiss Statement” 79, also followed the same path. Whereas evidence of RC should be shown in the decreased effectiveness of a given BI to prevent HIV transmission, most of the literature aiming to find and evaluate evidence of RC, primarily concern behavioural changes. A meta‐analysis 80 was undertaken aiming to determine if ART use was associated with changes in “unprotected” sex and STI diagnoses. Among 56 studies, condomless sex was found to be lower in participants receiving ART compared to those who were not (OR: 0.73 (95% CI: 0.64 to 0.83); *p *<* *0.001). Among 11 studies, STI diagnoses were found to be lower among participants receiving ART compared to those who were not (OR: 0.58 (95% CI: 0.33 to 1.01); *p *=* *0.053).

As a BI, PrEP has shown to be a viable method for those that do not systematically use condoms, ineffectively use other risk reduction strategies (RRS), or wish to have an extra layer of protection 81, 82. The demonstrated efficacy and effectiveness of PrEP among other KP, which led to expanding WHO PrEP recommendations, has been followed by numerous studies aiming to evaluate “RC” among PrEP users, some of which have been analysed in systematic reviews and meta‐analyses. STIs have been a major focus of these studies. While STIs are an obvious health concern and prevention strategies must be fully implemented in order to reduce their incidence, opportunities can be missed for those most at risk for HIV and other STIs if reflection on STI is restricted to the BI framework. First, because BI do not aim to reduce STI but HIV incidence. Second, because even if a same behaviour, CLS, leads to HIV and other STIs, the underlying psycho‐social mechanisms to prevent the former and the latter are different 27. STIs do not represent for individuals the same health concern as HIV, and the information, motivation and skills required to mobilise to prevent STIs are therefore different.

In a systematic review and meta‐analysis of the effectiveness of oral PrEP among at‐risk populations, sexual behaviour (defined as condom use and number of sexual partners, and used to identify the presence of RC) was studied as an outcome in addition to HIV infection, adverse events, and antiretroviral drug resistance 83. This analysis found that PrEP effectively protected against HIV infection across all populations. Although the authors found no evidence of RC with PrEP, and no evidence of RC in open‐label extension (OLE) studies which are more likely to show “real‐world use,” they caution that study participants benefited from behaviour counselling and were previously trial participants 83.

A systematic analysis of OLE and demonstration studies investigated the effect of PrEP use on SRB 84. While the authors rightly excluded studies that measured beliefs about PrEP use and/or predicted future behaviour, increase in “risky sexual behaviours” and “risk compensation” are used synonymously. “RC” was measured by using several outcomes, however, due to inconsistency across the studies in the measures of CLS and number of condomless partners, meta‐analysis was limited to STI diagnosis. Although there is evidence to suggest that an increase in number of CLS partners and general decline in condom use, this may be restricted to the proportion of MSM who already reported these behaviours 84.

The impact of PrEP use on SRB and RRS has also been examined in qualitative studies. Among 41 participants of the PROUD PrEP study 81, only half of them declared an increase in “risk taking behaviour.” The participants reported using various RRS before using PrEP (e.g. strategic positioning, sero‐sorting, PEP use), however, all reported (some) CLS. Overall, given inconsistent condom use and situations and contexts that may lead to increased risk taking, participants declared that PrEP filled a prevention gap or added another layer of protection for participants already at high risk 81.

A qualitative sub‐study conducted with iPrEx OLE participants 27 found that, in opposition to feelings of worry and concern regarding HIV infection that pervaded respondents’ lives, PrEP enabled to replace them with feelings of safety. For participants not using condoms prior to PrEP, thinking of a “PrEP‐as‐condom‐replacement theory” had no sense. For those using condoms and willing to use PrEP to engage in CLS, did not actually engage in CLS. More interestingly, respondents reporting sexual behavioural changes (going “crazy”) declared that the possible emergence of a STI was a reminder of PrEP's limits 27. Changes were therefore more emotional than behavioural.

Recently, Holt and Murphy 23 have introduced the concept of community‐level RC in the context of PrEP in which “changes in risk perceptions and behaviour (could occur) as a result of increased optimism about avoiding HIV among people not directly protected by PrEP.” However, due to increased PrEP uptake and consistent PrEP use among PrEP users, protection at the community‐level actually increased (reduction of HIV incidence). They propose monitoring changes in sexual behaviour in addition to attitudes to PrEP and perceived HIV risk. This could measure HIV “prevention optimism” defined as “the belief that it is easier to avoid HIV infection or transmission because of PrEP and that it is more acceptable and safer to engage in condomless sex because the risk of HIV is perceived to be reduced” 23. Further research is needed to explore the impact of “optimism,” particularly among non‐PrEP users.

### 2.3 PrEP: a concern or an opportunity for STI control?

PrEP is a significant step forward in the fight against HIV, not only for its impact on HIV transmission, but also its opportunity to increase the frequency of HIV and other STIs testing, to promote early diagnosis and treatment of HIV and other STIs. According to one modelling study, high PrEP coverage among MSM could lead to an important decline in STI incidence, largely attributed to routine testing which allows early detection and treatment of asymptomatic STIs 85. PrEP also has the potential to alleviate fears of HIV, to allow for a more fulfilling sex life 26, 27, and to empower individuals to protect themselves and others 86. Adapted and quality counselling around PrEP, sometimes community‐based, may be a favourable environment to have a discussion on sexual behaviour, drug use and other sexual health needs 28, 87, 88.

Several studies, however, have shown barriers on the part of medical providers to have such discussions 87, 89, and on the part of patients 90, 91 to share information regarding their sexual behaviour. Behavioural changes associated with BI need to be studied, however, there is still a major health issue: reaching, informing, testing, treating and empowering individuals, in order to integrate them into a preventive health path, not only for HIV but also for other STI.

Peer‐led counselling, offered in the ANRS‐Ipergay 4 and currently offered in the ANRS‐Prevenir study 92 by the French community‐based organisation AIDES, moves away from a “curative health system” perspective in which health consultations are driven by symptoms, towards a health path for HIV‐negative individuals that addresses overall sexual health based on the individual needs at a given point in life 88. From the perspective of PrEP users, peer counsellors use both their personal and community experience to inform and discuss the spectrum of prevention methods and how they may fit with individual needs. Building individual capacity to evaluate personal risk, and thus, empower PrEP users to find prevention strategies that meet their needs for a satisfying sexual life, can potentially have lasting effects, regardless of the duration of PrEP use. Although limited, longitudinal data on PrEP use has shown important decreases in retention over time 93, 94. Changes in sexual behaviours, perceived HIV risk, financial cost, adverse effects and problems related to adherence have been identified as reasons for PrEP discontinuation 93, 95. It is therefore increasingly important to address the fact that PrEP users may not be lifetime users and to put individuals on a preventive health path that is sustainable after PrEP discontinuation. Current PrEP studies should explore this issue to find potential solutions to minimize HIV and STI risk when individuals choose to no longer use PrEP.

Global rates of STI, which were rising before PrEP 30, remain a concern. While rising STI rates among PrEP users may be partially explained by increased testing in multiple anatomic sites within the context of PrEP follow‐up, other bio‐behavioural interventions, in addition to information, counselling and notification, must be explored. Over time, it is possible that repeat STI testing may result in a change of behaviour, particularly among those with high‐risk behaviours who may come to realise the limits of PrEP (e.g. repeat STIs) 27 and therefore may implement or return to other prevention methods.

New interventions should systematically be accompanied by measures to better inform on STIs, to reinforce individual perception of STI risk and to promote behavioural changes adapted to individual needs. These behavioural changes could result in condom use for some individuals, however, there are other interesting alternatives such as partner notification or BI for STIs. Recent studies on the prophylactic use of doxycycline for bacterial STIs have shown promising results post‐exposure 96 and used daily 97, but remain to be confirmed in studies with longer follow‐up 98. Use of doxycycline may be particularly pertinent among PrEP users who experience recurrent STIs; a recent analysis has shown that among MSM PrEP users, 25% participants accounted for a little more than three‐quarters of all STIs 99.

Such an integrated sexual health approach has a lot to learn from the PrEP model, which could become a gold standard in handling prevention. The PrEP model needs to be developed and expanded not only for those at risk for HIV, and among them, mostly for MSM, but also for all the populations, which could also prevent STIs. Women, migrants, transgender individuals, drug users could take benefit of a comprehensive health offer (as with PrEP).

If we want this to become a reality several conditions are needed. First, work with health‐care providers is needed. In order not to limit prevention options of patients, non‐judgmental discussion on sexual behaviour, and drug use, has to be ensured. Improving the patient‐provider relationship can be key to moving away from RC focus to a positive and integrated sexual health approach.

Second, medical practice and HIV prevention research will benefit from knowledge from other disciplines and methods. For example, qualitative studies can provide new and complementary information to already existing data. Additionally, a more critical approach to the theories or concepts exported from other fields would allow for a more efficient response to eliminate the epidemic and respond to the health needs of KP.

Finally, effective STI control will not be possible without political will, corresponding funding and implication of all stakeholders to test interventions such as partner notification, integration of sex education programmes in schools, or legislative changes regarding antibiotic treatment among others 30.

## 3 Conclusions

Effective BI for HIV and STIs have been plagued by debates of RC for centuries. The concept of RC, stemming from the field of road safety, has been the subject of theoretical controversy and its use has been reasonably questioned. And yet, RC remains a frequent argument to justify moral judgements against the availability and provision of prevention methods for vulnerable populations who already experience stigma and discrimination 100. Unsurprisingly, PrEP and its possible large‐scale implementation has also been discussed within the framework of RC potentially undermining its efficacy. Would the availability of an effective HIV vaccination prompt the same debates?

Gaps to improve and guarantee access to testing, treatment and to reach an undetectable viral load for KP are a harsh reality, which means that the end of the HIV epidemic will not happen anytime soon. Lack of access to HIV/STI treatment and prevention is deeply linked to the shame associated with them and to the stigma and discrimination that those with the disease have to face from some health providers. For these reasons, the full range of existing prevention options has to be made available. With the information and support provided by healthcare providers, and by community stakeholders, individuals must have the opportunity to choose the prevention method(s) that best respond to their health needs at a given point of their (sexual) life and thus protect themselves. From a human rights perspective, BI access should not be barred based on the presence (absence) of STIs or changes in sexual behaviour 28. Finally, the role of community‐based stakeholders cannot be overlooked in increasing knowledge regarding sexual health and the empowerment of populations deemed “at risk” to identify and adapt prevention strategies that best fit their needs.

HIV and STIs cannot be thought and addressed in a social vacuum 26, 101. Interdisciplinarity, community perspectives and long‐term evidence from PrEP cohorts are needed to disentangle the effects of the combination of different BI that coexist with societal changes that have an impact on individual and community behaviours and social representations of sex, sexual orientation and experience of STIs, including HIV. Despite proven efficacy and effectiveness of PrEP, scientific literature seems to have been more concerned on how PrEP could “increase risk” instead of on how it reduces it or on how PrEP could lead to the empowerment of individuals regarding sexual health 27, 28. Science, working hand‐in‐hand with communities, can dramatically improve the response not only to HIV but also to other STIs by implementing and assessing adapted interventions that are based on individual health needs.

## Competing interests

DRC and RMD declare no conflicts of interest. JMM is on the advisory boards for Gilead Sciences, Merck and ViiV.

## Authors’ contributions

DRC, RMD and JMM, discussed key ideas and concepts forming the basis of this debate article. RMD and DRC wrote the manuscript. All authors reviewed and approved the final version.
